# The Highly Selective and Near-Quantitative Conversion of Glucose to 5-Hydroxymethylfurfural Using Ionic Liquids

**DOI:** 10.1371/journal.pone.0163835

**Published:** 2016-10-06

**Authors:** Sanan Eminov, Agnieszka Brandt, James D. E. T. Wilton-Ely, Jason P. Hallett

**Affiliations:** 1 Department of Chemistry, Imperial College London, London, SW7 2AZ, United Kingdom; 2 Department of Chemical Engineering, Imperial College London, London, SW7 2AZ, United Kingdom; Queen's University Belfast, UNITED KINGDOM

## Abstract

A number of ionic liquids have been shown to be excellent solvents for lignocellulosic biomass processing, and some of these are particularly effective in the production of the versatile chemical building block 5-hydroxymethylfurfural (HMF). In this study, the production of HMF from the simple sugar glucose in ionic liquid media is discussed. Several aspects of the selective catalytic formation of HMF from glucose have been elucidated using metal halide salts in two distinct ionic liquids, 1-butyl-3-methylimidazolium chloride and 1-butyl-3-methylimidazolium hydrogen sulfate as well as mixtures of these, revealing key features for accelerating the desired reaction and suppressing byproduct formation. The choice of ionic liquid anion is revealed to be of particular importance, with low HMF yields in the case of hydrogen sulfate-based salts, which are reported to be effective for HMF production from fructose. The most successful system investigated in this study led to almost quantitative conversion of glucose to HMF (90% in only 30 minutes using 7 mol% catalyst loading at 120°C) in a system which is selective for the desired product, has low energy intensity and is environmentally benign.

## Introduction

The future development of the biorefinery concept will require efficient routes for the conversion of biomass to useful platform chemicals.[1, 2] Lignocellulosic biomass represents a promising renewable feedstock for commercial large-scale biorefining, as lignocellulose is distributed widely across the planet and can be grown on a billion ton scale.[2] Biomass based on agricultural by-products (e.g. wheat straw, sugarcane bagasse, corn stover) or crops, which do not rely on the use of arable land (e.g. *Miscanthus*, switchgrass, tree wood), avoid competition with food production and result in higher net CO_2_ emission savings, rendering this approach to sustainable fuels and chemicals extremely promising. In addition to reducing reliance on fossil fuels for energy, biorefining offers an opportunity to secure and diversify material supply chains and further reduce our carbon footprint as we turn toward a bio-based economy and potentially ‘carbon neutral’ manufacturing. However, processing of lignocellulosic biomass feedstocks is still difficult and expensive, sometimes relying on hazardous and energetically demanding pretreatment routes to separate lignin from cellulose,[3] such as large quantities of acidic aqueous or volatile organic solvent systems.[4–6] Such systems also suffer from low conversions and high waste generation (usually from acid neutralization). Subsequent sugar and biofuel production relies on slow biocatalytic transformations (enzymatic saccharification and microbial fermentation)[7] that generate additional aqueous waste that requires treatment. Ionic liquids (ILs)[8] are being developed as alternative, potentially more commercially attractive and low emission media for separating cellulose from lignin due to their unique chemical and solvent characteristics,[9] a process that is critical for further functionalization of the fractionated biopolymers.[9–12] It should be noted that, for the benefits of these solvent systems (high separation efficiency, negligible vapor pressure) to outweigh the demands of their synthesis, recycling and reuse will be a necessary condition of their use.

The U.S. Department of Energy has identified 5-hydroxymethylfurfural (HMF) as a major platform chemical that could be derived from lignocellulosic biomass on a large scale.[13] HMF is a key intermediate for a range of chemicals, such as 2,5-furandicarboxylic acid (FDCA)[14] which can be used to make renewable polymers[15] and for liquid transport fuels, such as 2,5-dimethylfuran, which has 40% higher energy density than ethanol and similar fuel performance to conventional gasoline.[16] The potential of HMF as a synthetic building block is now widely recognized, which has led to a surge in the amount of research utilizing HMF as a feedstock.[17]

**Fig 1:**
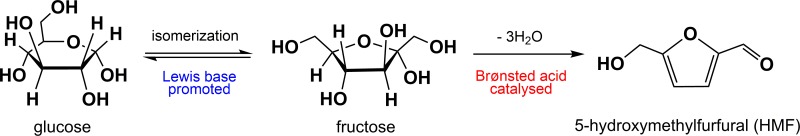
Glucose conversion to 5-hydroxymethylfurfural (HMF).

A number of side-reactions occur during or after HMF transformation, leading to formation of levulinic acid, formic acid and humins.[18, 19] These can reduce the yields substantially. For example, it is has been shown that the HMF product combines with the remaining monosaccharide substrate or an intermediate to produce water-insoluble humins under acidic conditions.[20] Despite these challenges, HMF can now be produced with sufficient selectivity and yield from fructose for it to become a nascent industrial process. Conversion of glucose to HMF, however, is much more challenging than fructose dehydration, as the conversion of glucose is known to proceed through a two-step mechanism involving two steps with distinct needs regarding the catalytic environment: (1) the isomerization of glucose to fructose followed by (2) dehydration to HMF (Fig 1).[16] The former reaction is catalyzed by Lewis base functionalities, while the latter reaction is promoted by Brønsted acids.[21] This tandem reaction system makes it more difficult to control the side reactions and achieve satisfactory selectivity and HMF yields, particularly in aqueous media. High selectivity in aqueous systems is further hampered by the formation of side products from HMF which involve addition of water (*e*.*g*. levulinic acid). Solubility of HMF can also be an issue at high sugar loadings in aqueous systems, leading to byproducts formed between substrates (sugars) or intermediates and product (HMF) leading to insoluble byproducts (humins). This has led some researchers to explore removing HMF from the aqueous phase (which contains the sugars) using immiscible organic solvents, thereby reducing humin formation.[22]

Tuning the acidity/basicity balance has been identified as a key factor to improving yield and selectivity of the glucose to HMF transformation. Support for this was recently obtained in an aqueous system using bifunctional sulfated zirconia catalysts with tunable acid-base properties.[21, 23] The addition of 0.1 M HCl to an AlCl_3_–H_2_O/THF biphasic system has also been reported to result in an 11% increase (to 62%) of HMF yield from glucose, further supporting the need to balance Lewis and Brønsted effects to improve the selectivity of glucose conversion.[24]

The potential for selectively making HMF from glucose using ionic liquids (ILs) as solvents was first demonstrated by Zhao *et al*.[25] This study assessed the ability of various metal salts to convert glucose to HMF in [C_4_C_1_im]Cl and found that chromium salts were the most active catalysts, providing 70% HMF yield at 100°C in 3 hours with CrCl_2_. This is better than most yields obtained in aqueous systems. The superiority of chromium salts over other metal species was subsequently verified by other groups.[26–30] Zhao also reported good yields of HMF from glucose, though the reason for the high Cr activity is not well understood.

Although this system is very effective, perceived toxicity of chromium is a key issue for commercial application. The reputation for toxicity of chromium is largely based on Cr(VI) compounds, while trivalent chromium(III) is actually an essential trace metal required for the formation of glucose tolerance factor and for insulin metabolism. Indeed, Cr(III) is widely used as a nutritional supplement for humans and animals.[31] Additionally, the risk of exposure to metal salts dissolved in ionic liquids is extremely low as their removal from the system is very difficult due to the well-known solvating power of ILs.

**Fig 2:**
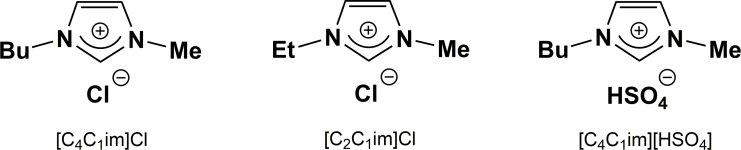
Ionic liquids used for the dissolution and breakdown of biomass in this work.

Additional reports of HMF synthesis from glucose in ILs used different catalysts but again predominantly employed imidazolium chloride ionic liquids, in which carbohydrates are highly soluble, mostly [C_4_C_1_im]Cl and [C_2_C_1_im]Cl (Fig 2).[27–30, 32] For example, a variety of zeolite catalysts were investigated in [C_4_C_1_im]Cl, with an Hβ-zeolite consisting of a unique BEA structure and a moderate Si/Al ratio of 25 possessing the highest catalytic activity (50% HMF yield at 150°C in only 50 min).[33] In order to improve the catalytic performance of β-zeolites, the effects of calcination and steam treatment on the structure of Al atoms in the framework and acid properties were examined in detail.[34] With sufficient Lewis acid sites available, the bifunctional catalyst system demonstrated 55% selectivity for HMF from glucose. In another report, a tin-containing silica molecular sieve (Sn-MCM-41) was found to act as a bifunctional heterogeneous catalyst for the conversion of glucose to HMF in [C_2_C_1_im]Br, with 70% yield at 110°C after 4 h.[35] The resulting yields and selectivities for these IL systems can be compared with organic (sometimes biphasic) solvent systems. For example, this 70% yield is much higher than that reported for several mesoporous, ZrO_2_ containing MCM-41 silicas used in a biphasic water/MIBK solvent systems (23% HMF yield).[36] A similar HMF yield was obtained using mesoporous tantalum oxide as a catalyst, also in biphasic water/MIBK.[37] Using a glucose:catalyst weight ratio of 3:1, glucose conversion of 56% and an improved HMF yield of 33% was achieved at 170°C in one hour.[37] Dumesic *et al*. reported[22] higher yields of 62% with a Lewis acid (AlCl_3_) and Brønsted acid (HCl) catalyst combination in biphasic water/2-*sec*-butylphenol.

Ionic liquids have also been explored as additives/co-solvents rather than as the sole liquid medium for the reaction. For example, addition of 1-hydroxyethyl-3-methylimidazolium tetrafluoroborate as a catalyst (rather than as a solvent) led to HMF yields as high as 67% after 1 h at 180°C in dimethylsulfoxide.[38] In homogeneously catalyzed systems, the combination of CrCl_3_·6H_2_O (10 mol%) and B(OH)_3_ (20 mol%) was reported to act as a double catalyst in [C_4_C_1_im]Cl to provide an HMF yield of 79%, achieved at 120°C in only 30 min.[39] In 1:1 [C_6_C_1_im]Cl/water biphasic mixtures, HMF yields of 53% were obtained within just 10 min at 200°C in the presence of ZrO_2_.[40] It is clear that different ionic liquid systems (such as those shown in Fig 2) can exert a substantial effect on catalyst activity and byproduct formation.

Bearing in mind that ILs can be tuned or designed to suit a certain application, the mildly acidic ionic liquid 1-butyl-3-methylimidazolium hydrogen sulfate, [C_4_C_1_im][HSO_4_] (Fig 2) was recently employed as the solvent system for the conversion of fructose to HMF.[26] Only a low loading of chromium(III) chloride was required in order to achieve an excellent HMF yield of 96% in 3 hours at 100°C without any detectable trace of the common byproducts, levulinic acid, formic acid or humins (insoluble polymers formed from reaction of HMF with the monosaccharide). Since the dehydration of fructose is known to be favored by acidic conditions,[29, 41] the choice of a mildly acidic IL seemed a logical choice for promoting fructose conversion, while the non-aqueous ionic liquid solvent appeared to suppress side reactions (which often involve the addition of water). This imidazolium-based ionic liquid shares the same anion ([HSO_4_]^-^) with a family of ionic liquids that have recently been identified as true low-cost options ([HNEt_3_][HSO_4_] and [C_1_Him][HSO_4_]), with a similar cost to traditional organic solvents such as acetone.[42] One aim of this study was therefore to investigate whether the promising results based on fructose could be extended to other sugar substrates, and in particular, to glucose.

In addition to the delicate interplay between acidity and basicity during glucose to HMF transformation in all solvent systems, including ILs, the coordinating ability of the IL anion has been shown to be an additional significant factor when metal catalysts are used to convert glucose to HMF. For example, the Lewis acidic metal catalyst SnCl_4_ has been used in ILs with a [C_4_C_1_im]^+^ cation and a range of anions.[27] It was found that the highest activity was achieved when BF_4_^-^ was the IL anion, while ILs with more coordinating anions (including Cl^-^) demonstrated lower yields. This is consistent with the view that coordinating anions compete with the sugar substrate for catalyst sites (metal-sugar complexation is an important step in the fructose-to-HMF dehydration[16, 25, 28]). Thus Lewis basic anions that are strongly coordinating can inhibit the formation of HMF.[27]

In contrast to the simple acid-catalyzed mechanism for HMF production from fructose, these requirements provide conflicting information as to which ionic liquid type would be optimal for HMF formation from glucose. As such, it was unclear prior to this study whether a Lewis basic and a highly coordinating IL (such as [C_4_C_1_im]Cl) or a Brønsted acidic IL (such as [C_4_C_1_im][HSO_4_]) would favor glucose transformation in the presence of metal catalysts. The study presented here provides insights into this process and applies them to achieve near quantitative and selective conversion of glucose to HMF in a short reaction time.

## Results and Discussion

### Catalyst and ionic liquid selection

As mentioned above, a yield of 96% HMF from fructose was obtained in a previous study employing hydrogen sulfate-based ILs, showing that these ILs are excellent at speedily and selectively dehydrating fructose.[26] The same ILs and conditions were applied with glucose as a substrate in order to compare HMF yields from glucose to those from fructose. A high HMF yield would suggest that the fructose dehydration step is the rate determining step, or improves the selectivity by reducing humin and levulinic acid formation. Several metal catalysts and two temperatures were examined in [C_4_C_1_im][HSO_4_]. Samples were collected after 0.5, 1, 3 and 24 h and analyzed by ^1^H NMR spectroscopy (Table 1). After 0.5 h, formation of HMF was not observed under any conditions. When using CrCl_3_ as catalyst at 80°C a (non-isolated) HMF yield of less than 5% was observed after 3 h (Table 1, entries 1 and 2), which is poor compared to the 96% yield obtained from fructose in the same reaction system.[26] Without any catalyst, no HMF could be detected even after 3 h (Table 1, entry 6). After 24 h, only 15% HMF yield was observed for the Cr catalysts and only 3% for all other metals tested. These results indicate that, while the Cr(III) catalyst appears a preferred choice for glucose conversion to HMF in this ionic liquid, the [C_4_C_1_im][HSO_4_] IL system does not provide the optimal environment at 80°C.

**Table 1:** Yield of HMF from glucose in [C_4_C_1_im][HSO_4_] at 80°C and 100°C.

Entry	Metal salt	Temp.	Substrate		0.5 h	1 h	3 h	6 h	24 h	48 h		72 h
**1**	CrCl_3_·6H_2_O	80°C		glucose	0	<3	<5	-	15		-	-
**2**	CrCl_2_	80°C		glucose	0	<3	<5	-	15		-	-
**3**	K_2_PtCl_4_	80°C		glucose	0	0	0	-	<3		-	-
**4**	NiCl_2_	80°C		glucose	0	0	0	-	<3		-	-
**5**	ZnCl_2_	80°C		glucose	0	0	0	-	<3		-	-
**6**	no catalyst	80°C		glucose	0	0	0	-	<3		-	-
**7**	CrCl_3_·6H_2_O	100°C		glucose	0	15	15	15	33		15	10
**8**	CrCl_2_	100°C		glucose	0	15	15	15	27		33	10
**9**	no catalyst	100°C		glucose	0	0	0	0	<15		<15	0
**10**	CrCl_3_ 6H_2_O	100°C		galactose	0	<3	15	-	-		-	-
**11**	CrCl_2_	100°C		galactose	0	<3	15	-	-		-	-
**12**	no catalyst	100°C		galactose	0	0	0	-	-		-	-

0.1 g glucose in 0.7 g [C_4_C_1_im][HSO_4_]; 7 mol% catalyst where present. Non-isolated yields shown.

Among the catalysts tested, chromium chloride compounds promoted the highest HMF yields in [C_4_C_1_im][HSO_4_], in line with earlier fructose results.[26] Hence further catalytic studies were restricted to Cr-based catalysts. Attempts were made to raise the HMF yield by increasing the temperature to 100°C under identical catalyst and glucose loadings. However, HMF formation was not observed after 0.5 h, even at the elevated temperature (Table 1, entries 7–9). A 15% yield was obtained after 3 h, which was only moderately more than was found at 80°C (Table 1, entries 7 and 8).

In order to probe whether the yields can be influenced by changing the isomerization step, the CrCl_3_/[C_4_C_1_im][HSO_4_] catalytic system was applied to galactose, which is a C4 epimer of glucose. Table 1 demonstrates that the conversion of galactose to HMF showed essentially the same yields as the transformation of glucose to HMF (Table 1, entries 10–12). Since the galactose system must undergo a similar pathway to the glucose/fructose isomerization before dehydration, the similarity of these yields supports our findings that this transformation is limiting the HMF yield.

It is important to note that in all of our experiments any standing concentration of fructose as an intermediate was below the detection limit of the NMR method employed (also S1 Fig in the ESI).[43] In the later work using chloride-based ILs, the presence of fructose as an intermediate in the reaction pathway was probed using both NMR and HPLC (ESI, S2 Fig) and fructose was detected, but not in the presence of hydrogen sulfate anions.

Once the variation of catalysts and temperature had been investigated, additional data were collected to examine the effect of longer reaction times on the conversion of glucose to HMF (Table 1, entries 7–9). It was hoped that this would help elucidate whether the low yield was solely due to slow kinetics, or if HMF side reactions (to form levulinic acid and/or humins) would occur over extended reaction times. An HMF yield of 33% was obtained using CrCl_3_·6H_2_O at 100°C after 24 h (Table 1, entry 7), an increase from 15% after 6 h. However, the yield of HMF dropped thereafter over longer reaction times with only 10% remaining after 72 hours. Analysis by ^1^H NMR spectroscopy revealed that the formation of levulinic acid was the main reason for this decrease in yield. Using CrCl_2_ also resulted in the same 10% yield after 72 hours, although this reaction appeared to proceed more slowly (Table 1, entry 8).

### The effect of ionic liquid mixtures

Since [C_4_C_1_im]Cl is capable of enhancing the isomerization of glucose to fructose through the Lewis basicity of the Cl^-^ anions, and [C_4_C_1_im][HSO_4_] accelerates the conversion of fructose to HMF, it was thought that it may still be possible to obtain an optimum mixture with which to accomplish the conversion of glucose to fructose, and then fructose to HMF (Table 2).

**Table 2:** Yield of HMF from glucose in different ionic liquids at 120°C.

Entry	Ionic liquid	5 min	10 min	15 min	20 min	30 min	1 h	3 h
**13**	[C_4_C_1_im]Cl	51	61	65	65	65	65	65
**14**	[C_4_C_1_im][HSO_4_]	-	-	-	-	<3	6	21
**15**	[C_4_C_1_im]Cl + [C_4_C_1_im][HSO_4_] (1:1)	-	-	-	-	19	19	19
**16**	[C_4_C_1_im]Cl + [C_4_C_1_im][HSO_4_] (1:3)	-	-	-	-	23	23	23
**17**	[C_4_C_1_im]Cl + [C_4_C_1_im][HSO_4_] (3:1)	-	-	-	-	21	21	21
**18**	[C_4_C_1_im]Br	29	35	35	35	35	-	35

0.1 g glucose in 0.7 g ionic liquid; 7 mol% CrCl_3_·6H_2_O catalyst, 120°C. Yields shown are non-isolated.

As a benchmark, the reaction was performed in pure [C_4_C_1_im]Cl (7 mol% CrCl_3_·6H_2_O, 120°C, 3 hours)[25] to provide a maximum HMF yield of 65% (Table 2, entry 13), which is very similar to published results for this system using CrCl_2_ as the catalyst.[25] The experiments were carried out at 120°C to have sufficient fluidity in the relatively high melting [C_4_C_1_im]Cl. An equimolar mixture of [C_4_C_1_im][HSO_4_] and [C_4_C_1_im]Cl was then tested using the same catalyst system under otherwise identical conditions, however, this mixture did not perform well; an HMF yield of only 19% was obtained after 30 min-3 h (Table 2, entry 15).

**Fig 3:**
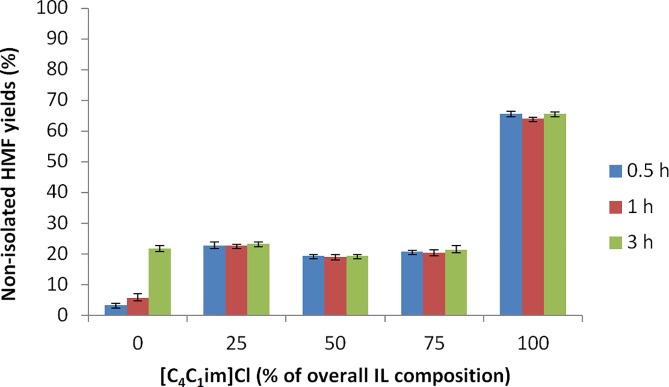
HMF yield from glucose in pure [C_4_C_1_im]Cl and mixed with [C_4_C_1_im][HSO_4_] at 120°C. 0.1 g glucose in 0.7 g ionic liquid, 7 mol% CrCl_3_·6H_2_O catalyst. Reported yields are non-isolated.

HMF yields (19–23%) very similar to the yield employing CrCl_3_·6H_2_O in pure [C_4_C_1_im][HSO_4_] were also obtained using other ionic liquid mixtures (containing 25%-75% [HSO_4_]^-^), with pure [C_4_C_1_im]Cl leading to the highest yield (Table 2, entries 13–17 and Fig 3) by a substantial margin. These results suggest that the presence of any [C_4_C_1_im][HSO_4_] lowers the conversion of glucose to HMF, and strongly suggests that the [HSO_4_]^-^ anion impedes the glucose-fructose isomerization. It thus appears that the [C_4_C_1_im][HSO_4_] ionic liquid, which promotes fructose to HMF conversions so successfully, is a poor medium for glucose conversion to HMF.

From this observation of low yield for the reaction in [C_4_C_1_im][HSO_4_], it can be inferred that the initial step of the transformation–the isomerization of glucose to fructose–is the more important catalytic step in the overall conversion of glucose to HMF. Furthermore, it is clear that the Lewis basic IL (containing the Cl^-^ anion) is more suitable than the Brønsted acidic solvent ([HSO_4_]^-^ anions). This is likely to be due to the formation of [CrCl_4_]^-^ in solution, which is capable of promoting the glucose isomerization.(28) In contrast, the non-coordinating [HSO_4_]^-^ ion cannot form such a complex, and therefore is probably less able to catalyze the glucose isomerization reaction, resulting in limited glucose fructose isomerization and hence low HMF yields. We can also infer that the [HSO_4_]^-^ forms a complex with CrCl_3_, and this complex must form preferentially over the [CrCl_4_]^-^ complex, otherwise hydrogen sulfate wouldn’t serve as a ‘catalyst poison’ even at low [HSO_4_]^-^ content as we observe here. Our results also confirm various hypotheses relating to mechanistic details in the conversion of glucose to fructose.

The importance of the [CrCl_4_]^-^ complex in the isomerization was established by the findings of Remsing and co-workers, who suggested that solvation of sugars in [C_n_C_1_im]Cl occurs through hydrogen bonding of the chloride ions in the solvent with the carbohydrate hydroxy groups.[44] NMR spectroscopy studies showed that the glucose starting material, when dissolved in [C_2_C_1_im]Cl, is predominantly an α-anomer.[16] They confirmed that little mutarotation (interconversion of the α- and β-anomers) was found to occur in pure [C_2_C_1_im]Cl, even after several hours at 80°C. However, in the presence of a catalytic amount of metal chloride salts, rapid mutarotation was observed, leading to an equilibrium mixture of anomers.[16]

It has also been suggested that HMF is formed through an acyclic mechanism involving a linear enediol,[45–48] produced during the isomerization of glucose to fructose. Glucose is also susceptible to competing reactions that lead to formation of by-products, such as dehydration to form non-furan cyclic ethers or C-C bond scission through a reverse aldol condensation.[46] Selective *in situ* isomerization to fructose is thus required to obtain high HMF yields from glucose.

As described above (Table 2, entry 13), this study has shown that a 65% HMF yield was possible after only 30 minutes, indicating a fast reaction. However, the yield did not increase further after 30 minutes (Table 2, entry 13) due to increased formation of levulinic acid as a byproduct (^1^H NMR spectroscopy). The duration of the reaction was then decreased to a matter of minutes in order to ascertain when the reaction had reached completion. It was discovered that the reaction reached completion after only 15 minutes (65% yield, Table 2, entry 13) in [C_4_C_1_im]Cl at 120°C, a substantially shorter duration than the standard 3 hours reported previously in the literature, even accounting for Arrhenius activation[25] (though faster times have been reported with microwave irradiation[28](28). In fact, a 51% yield was recorded after only 5 minutes of reaction time, which is reasonably close to the final yield of the reaction under these conditions. An alternative halide-based IL, [C_4_C_1_im]Br, was employed as a comparison, however, lower yields were obtained using this IL than for the chloride analogue (Table 2, entry 18) with 35% HMF yield being obtained after 10 minutes followed by no further change. This is likely due to differences in coordination power between the Cl^-^ and Br^-^ halide ions in the [CrX_4_]^-^ ion complexes.

**Fig 4:**
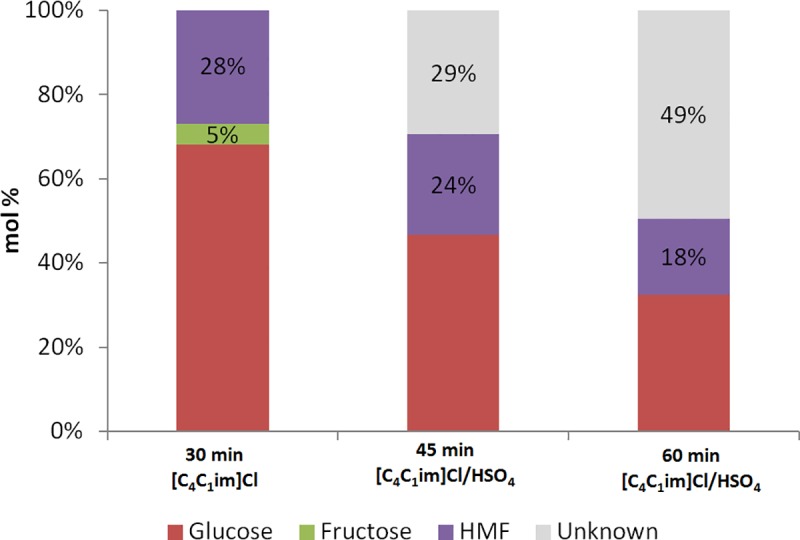
Yield of HMF from glucose at 90°C in pure [C_4_C_1_im]Cl and with subsequent addition of [C_4_C_1_im][HSO_4_], as determined by HPLC analysis. 0.1 g glucose in 0.7 g [C_4_C_1_im]Cl, 8.1 mol% CrCl_3_·6H_2_O catalyst. 0.3 g [C_4_C_1_im][HSO_4_] was added after 30 min. The mixture was heated again and samples were taken 15 min and 30 min later.

In order to further elucidate the effects of the IL anions on HMF production and byproduct formation, [C_4_C_1_im][HSO_4_] was added to a stirred solution of glucose in [C_4_C_1_im]Cl (with CrCl_3_·6H_2_O present). As the reaction was found to be too rapid at 120°C to perform this experiment reliably, a lower temperature (90°C) was used, which provided a manageable timeframe for the reaction. After 30 minutes at 90°C, the reaction mixture was analyzed by HPLC. This allowed not only the quantification of HMF formation but also the determination of glucose and fructose concentrations. Fig 4 shows the HMF yield after 30 minutes in pure [C_4_C_1_im]Cl to be 28% (consistent with the lower temperature). This decreased to 24% after introduction of [C_4_C_1_im][HSO_4_] and an additional 15 minutes stirring with exposure to the now mixed Cl^-^/[HSO_4_]^-^ ionic liquid solution. The HMF yield decreased further to 18% after 60 minutes total reaction time (and 30 minutes of exposure to the mixed solvent system). Examining the mass balance (Fig 4), it is clear that there was no mass loss (as assessed by HPLC) when using the initial [C_4_C_1_im]Cl solution, highlighting the excellent selectivity of the glucose to HMF transformation in this solvent system. However, in the mixed system, 29% (after 45 mins) and later 49% (after 60 mins) of the molecules could not be traced, which is consistent with the proposed formation of humins (levulinic and formic acid were not detected).

It is noteworthy that 5% fructose was detected after 30 minutes reaction in [C_4_C_1_im]Cl solution, but no fructose was detected after addition of [C_4_C_1_im][HSO_4_] to the system, likely due to rate differences in the dehydration between the two systems, in line with our previous results.[26] This is also consistent with the findings reported above and the proposed mechanism of glucose to fructose isomerization followed by dehydration, which appears to be valid not only in aqueous systems but also in ionic liquids. We conclude that while the dehydration reaction appears to be accelerated by the acidic [C_4_C_1_im][HSO_4_] IL, it also catalyzes the formation of HMF-glucose conjugates, as indicated by the apparent loss of HMF yield to humins when the [HSO_4_]^-^ anion is present. It is not apparent why these same humin conjugates do not form from HMF and fructose in this solvent.[26] The non-acidic [C_4_C_1_im]Cl does not appear to catalyse this humin formation to the same degree; indicating a superior selectivity toward HMF over humins for this IL compared to the more acidic [C_4_C_1_im][HSO_4_], again in contrast to the reported behavior for fructose as a substrate.[26]

Given that no humin formation was indirectly observed in the effectively quantitative formation of HMF from fructose in the CrCl_3_/[C_4_C_1_im][HSO_4_] system,[26] these results could suggest that humins form more readily from the reaction between HMF and glucose (or an intermediate of the glucose-fructose isomerization) than between HMF and fructose (at least in this system[49]). The suppression of isomerization of glucose to fructose by the addition of [HSO_4_]^-^ after 30 minutes (Fig 4) blocks this pathway to HMF. This leaves the HMF formed (28%) at that point to react with the glucose (67%) present, resulting in a decrease in the masses of both 15 minutes later and the concomitant formation of 29% lost mass, presumably humins.

These investigations provided further confirmation that the CrCl_3_/[C_4_C_1_im][HSO_4_] catalytic system, which had been successful for the production of HMF from fructose, was unsuitable for converting glucose selectively to HMF. The focus thus moved to investigating and improving the chromium catalyzed [C_4_C_1_im]Cl system popularized by Zhao,[25] and which is widely used for this conversion.

### Effect of temperature

**Fig 5:**
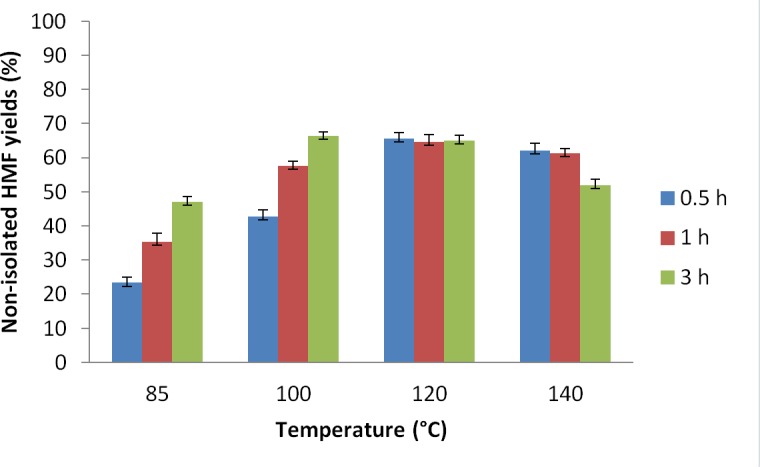
Yield of HMF from glucose as a function of reaction temperature from 85–140°C. 0.1 g glucose in 0.7 g [C_4_C_1_im]Cl, 7 mol% CrCl_3_·6H_2_O catalyst. Yields shown are not isolated. The lines are included solely as an aid to guide the eye.

This phase of the investigation commenced with the screening of different temperatures on the CrCl_3_/[C_4_C_1_im]Cl system for glucose to HMF conversion. At 85°C the reaction was sluggish, with only a 21% HMF yield observed after 30 min (Fig 5), rising to 48% after 3 hours. Increasing the reaction temperature to 100°C raised the yield to 65% after 3 h, but the reaction was still slow (43% yield after 0.5 h, Fig 5). Raising the temperature substantially, to 140°C, achieved a 65% yield of HMF after only 0.5 h (Fig 5). However, at this temperature, the yield dropped to 50% after 3 h due to the formation of humins. As mentioned above, the formation of these unknown compounds at high temperatures is attributed to the reaction between the HMF generated and the unreacted pyranose sugars (or intermediates) present.[50] As a result of these experiments, 120°C was selected as the best compromise temperature (Table 2, entry 13), allowing a rapid reaction and good yields, while avoiding substantial humin formation.

### Catalyst loading

In order to probe whether the Cr(III) catalyst loading chosen (7 mol%) was sufficient and whether catalyst poisoning in the reaction medium was a significant factor, the catalyst loading was increased. It was found that a fourfold increase in the catalytic loading (to 28 mol%) had a minimal effect on HMF production (61% after 0.5 h at 120°C) and so the 7 mol% loading was used as the standard loading for the remaining experiments. This result also suggests that Cr catalysts promote HMF formation through enhancement of the glucose-fructose isomerization, rather than by complex stabilization of HMF itself.

### Inhibition of byproducts

**Fig 6:**
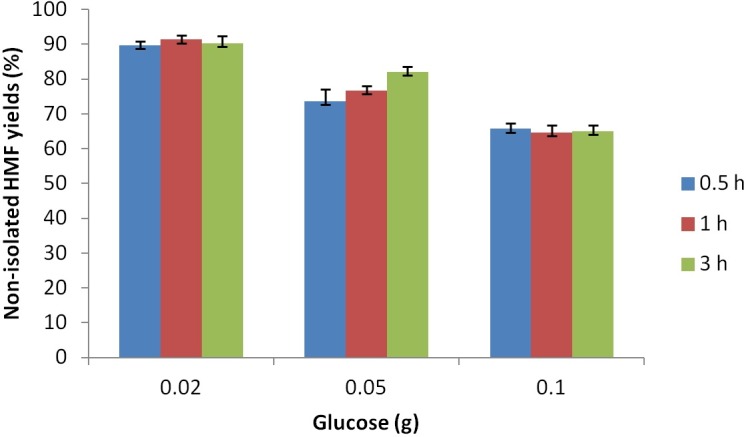
HMF yield from glucose as a function of substrate loading at 120°C. 0.7 g [C_4_C_1_im]Cl, 7 mol% CrCl_3_·6H_2_O catalyst. Reported yields are non-isolated. The lines are included solely as an aid to guide the eye.

The evidence from the mass balance data revealed the extent of humin formation, created from the reaction of HMF with the remaining sugar present. It was decided to explore the effect of reducing the sugar concentration, based on the hypothesis that the HMF formed would have less sugar with which to react and so reduce the generation of humins. The rapid nature of the reaction under the optimum conditions described above serves to limit the formation of humins as the amount of glucose present in the reaction medium is quickly reduced, preventing it from reacting (at least to some degree) with the HMF being generated. This is demonstrated in Fig 6, which shows that lowering the glucose loading from 0.1 g to 0.05 g and ultimately to 0.02 g led to HMF yields of 80% and 90%, respectively.

It can also be seen from Fig 5, that these high yields can be achieved in very short reactions times, as there was little or no increase in HMF yield beyond 30 minutes of reaction. To the best of our knowledge, this is the first report that glucose concentration can be manipulated to lower humin formation in ionic liquids, though this result is consistent with the findings of Dee and Bell.[50] This finding opens up the possibility of using reactor design to control glucose concentration in the reaction media, thereby enhancing HMF yields.

A potential limiting factor in the use of ILs for this transformation is separation of HMF from the IL solution, although recently it has been shown that HMF can be extracted with supercritical carbon dioxide.[51] Another issue is IL cost, if move away from the low-cost [HSO_4_]^-^ based salts.[42]

## Conclusion

The conversion of glucose to the established platform chemical, 5-hydroxymethylfurfural (HMF) is an important transformation within the biorefinery concept. Generating HMF from glucose rather than fructose brings us one step closer to the overall goal of using sucrose, starch and even cellulosic biomass to produce this polyfunctional intermediate. Our earlier work showed how [C_4_C_1_im][HSO_4_] was highly effective at producing HMF from fructose, however, the present study illustrates that this IL is not suitable for generating HMF directly from glucose. This can be traced to the need for an effective Lewis base to enable the isomerization of glucose to fructose, which is not readily available in the Brønsted acidic hydrogen sulfate-based IL. This study shows that addition of [C_4_C_1_im][HSO_4_] does not efficiently promote formation of HMF from glucose, in contrast to its effective role in fructose to HMF conversion. Instead, an IL with only Lewis basic Cl^-^ anions, such as [C_4_C_1_im]Cl, is required to form powerful coordinating species with the metal catalyst in solution, e.g. [CrCl_4_]^-^. These are then capable of promoting the glucose isomerization. Lastly, the high temperatures at which the reaction is typically performed (120°C) led to reaction of the HMF formed with unreacted sugars to generate humins, initially limiting the yield of HMF to around 65% using the CrCl_3_/[C_4_C_1_im]Cl system. This work has found that reducing the concentration of glucose leads to a (non-isolated) HMF yield of 90% in only 30 minutes (7 mol% catalyst at 120°C), without the need for further additives (e.g., boric acid[39]). Investigations are currently underway in our laboratories to adapt this approach to a flow process which operates at low concentrations of glucose. The ultimate goal will be to exploit the unique properties of ILs for both the pre-treatment and transformation of biomass to platform chemicals and fuels, ideally within one process.

## Experimental Section

General Comments. All experiments were carried out under aerobic conditions but with moisture excluded. The ionic liquids used, [C_4_C_1_im]Cl, [C_4_C_1_im][HSO_4_], and its precursor [C_4_C_1_im][MeSO_4_], were prepared using the slightly modified literature procedures detailed in an earlier report.[26] The metal salts were obtained from commercial suppliers. NMR spectroscopy was performed at 25°C using a Varian Mercury 300 spectrometer. All couplings are reported in Hertz.

### Catalytic formation of 5-hydroxymethylfurfural (HMF)

In each run, glucose (100 mg, 0.555 mmol) was added to a round bottom flask containing either [C_4_C_1_im]Cl (700 mg, 4.008 mmol) or [C_4_C_1_im][HSO_4_] (700 mg, 2.963 mmol). The catalyst (0.039 mmol, 7 mol%) under study (CrCl_2_, CrCl_3_∙6H_2_O, K_2_PtCl_4_, NiCl_2_, ZnCl_2_) was added to the flask. The flask was equipped with a reflux condenser and the reaction mixture was stirred at the desired temperature (80, 100, 120 or 140°C) for 24 hours and in certain cases for 72 hours. Samples were collected after various reaction times (see Tables 1 and 2) and analyzed by ^1^H NMR spectroscopy. HMF yields were calculated from the integration of HMF resonances against those of the ionic liquid [C_4_C_1_im]Cl itself (see ESI). The ionic liquid was considered to be an internal standard, as it has negligible vapor pressure. The distinct HMF resonances at 4.51 and 9.55 ppm in ^1^H NMR spectrum were chosen for calculation of the yields. Experiments were performed three times and an average taken. ^1^H NMR (400 MHz, DMSO) δ 9.55 (s, 1H, CHO), 7.50 (d, 1H, furan-CH, *J*_HH_ = 3.5 Hz), 6.61 (d, 1H, furan-CH, *J*_HH_ = 3.5 Hz), 5.57 (t, 1H, OH, *J*_HH_ = 6.0 Hz), 4.51 (d, 2H, CH_2_O, *J*_HH_ = 6.0 Hz) ppm.

### HMF production in system modified by sequential addition of another IL

Dry [C_4_C_1_im]Cl (704.5 mg, 4.03 mmol) was mixed with glucose (104.9 mg, 0.582 mmol) and chromium(III)chloride hexahydrate (12.6 mg, 0,047 mmol) in a two neck round bottom flask (25 mL) equipped with a stirring bar. The mixture was stirred at 90°C. After 30 min, two small samples were withdrawn for ^1^H NMR and HPLC analysis. The mixture was then cooled rapidly and [C_4_C_1_im][HSO_4_] (299.1 mg, 1.27 mmol) added. The mixture was then heated again and samples taken 15 min and 30 min later for ^1^H NMR and HPLC analysis.

### HPLC analysis

Approximately 20 mg (ca. 2–3 drops) of the reaction solution was added to pre-weighed centrifuge tubes (1.5 mL) with a known amount of water (ca. 400 mg). The samples were vortex-mixed until homogenous and centrifuged at maximum speed for 5 min. The supernatant was transferred to an HPLC vial with a 200 μL insert. HPLC analysis was performed on a Shimadzu Prominence instrument with refractive index (RI) and ultraviolet (UV) detector and cooled autosampler. The HPLC column used was an Aminex HPX-87H column, which was used with aqueous 10 mM sulfuric acid as eluent. The column temperature was 55°C, the flow rate 0.6 mL/min, the injection volume 10 μl and the run time 45 min. Four standard mixtures, each containing a different amount of glucose (10, 4, 2, 1 mg/mL), fructose (1, 0.4, 0.2, 0.1 mg/mL) and HMF (2, 1, 0.5, 0.1 mg/mL) were prepared. Sugars were identified and quantified using the refractive index signal, while the same was done for HMF using the 254 nm signal of the UV/Vis trace. Calibration curves with an R^2^ value of 0.9997 or better were obtained. The concentration in the HPLC samples was determined using the regression equations. Taking into account the dilution, the yields or conversions were then calculated.

## Supporting Information

S1 Fig Time course overlay of ^1^H NMR spectra of reaction solution from 0–60 minutes at 90°C. 0.1 g glucose in 0.7 g [C_4_C_1_im]Cl with 8.1 mol% CrCl_3_^.^6H_2_O. At 0 min, two glucose signals are prominent in the area relevant for quantification, while these peaks dissappear as the reaction progresses (probably due to fast exchange) and the 4.5 ppm HMF peak appears.
(TIF)

S2 Fig Sample HPLC chromatograms shown in time course overlay. Peak 1 –glucose; peak 2 –fructose; peak 3—HMF. The trace is from the RI detectorwhich shows both HMF and the sugars. The UV/vis trace was used for quantification of HMF due to its higher sensitivity.
(TIF)

S3 Fig Sample ^1^H NMR spectrum of the 30 minute reaction solution at 90°C. The mixture contained 0.1 g glucose, 0.7 g [C_4_C_1_im]Cl, 8.1 mol% CrCl_3_^.^6H_2_O. HMF signals appear at 7.5, 6.6, 4.9 and 4.5 ppm.
(TIF)

S4 Fig Sample ^1^H NMR spectrum of 0.1 g glucose in 0.7 g [C_4_C_1_im]Cl. (TIF)

S5 Fig Sample ^1^H NMR spectrum of 0.1 g fructose in 0.7 g [C_4_C_1_im]Cl. (TIF)

S6 Fig Sample ^1^H NMR spectrum of 0.1 g 5-HMF in 0.7 g [C_4_C_1_im]Cl. (TIF)

S7 Fig Sample ^1^H NMR spectrumof [C_4_C_1_im]Cl. (TIF)
