# Supplementary figures and images for: The Highly Selective and Near-Quantitative Conversion of Glucose to 5-Hydroxymethylfurfural Using Ionic Liquids
Source: PLoS One. 2016 Oct 6;11(10):e0163835. doi: 10.1371/journal.pone.0163835 (PMC5053443; doi:10.1371/journal.pone.0163835)

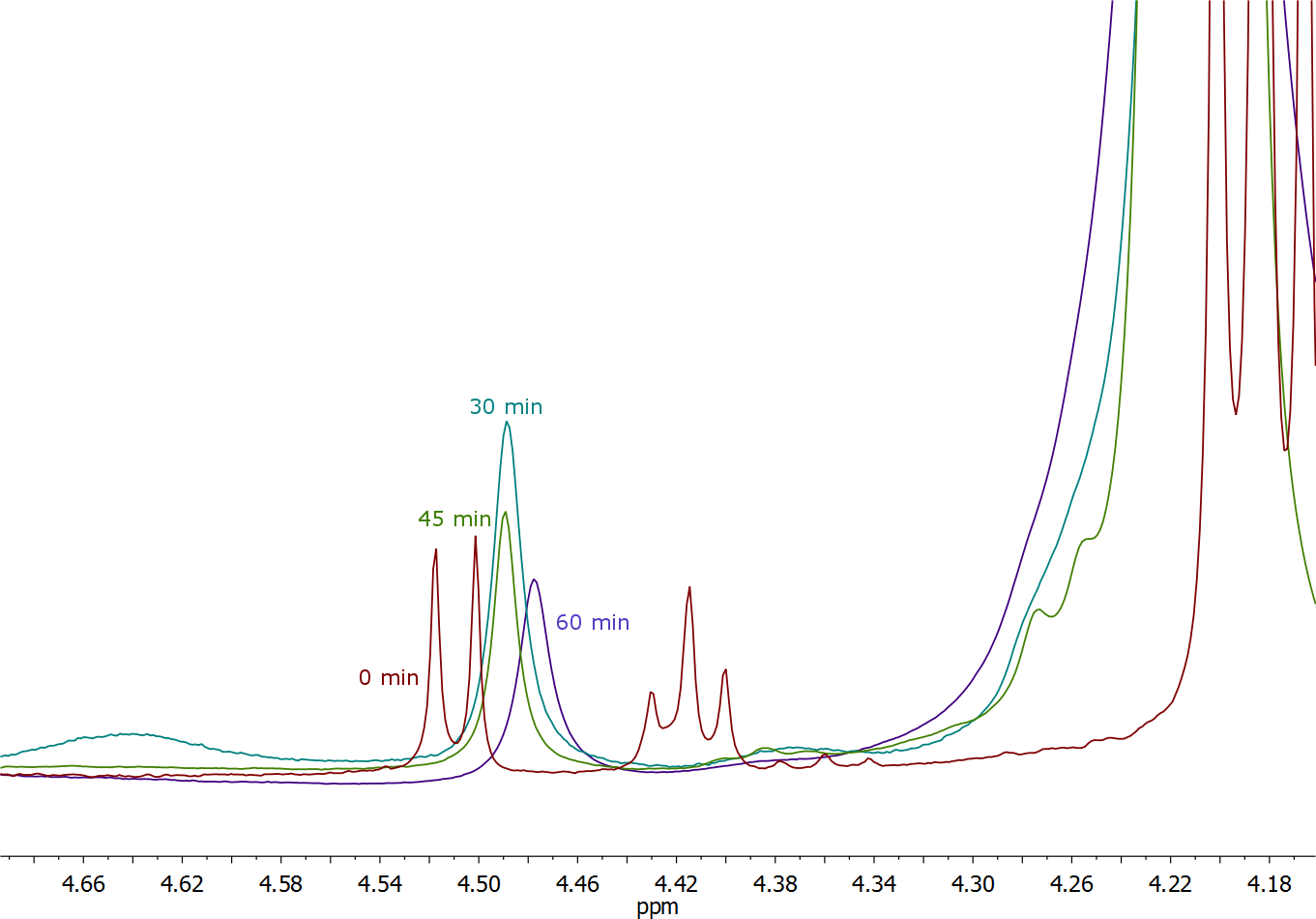

Supplement: S1 Fig — 0.1 g glucose in 0.7 g [C4C1im]Cl with 8.1 mol% CrCl3.6H2O. At 0 min, two glucose signals are prominent in the area relevant for quantification, while these peaks dissappear as the reaction progresses (probably due to fast exchange) and the 4.5 ppm HMF peak appears. (TIF) [file pone.0163835.s001.tif]

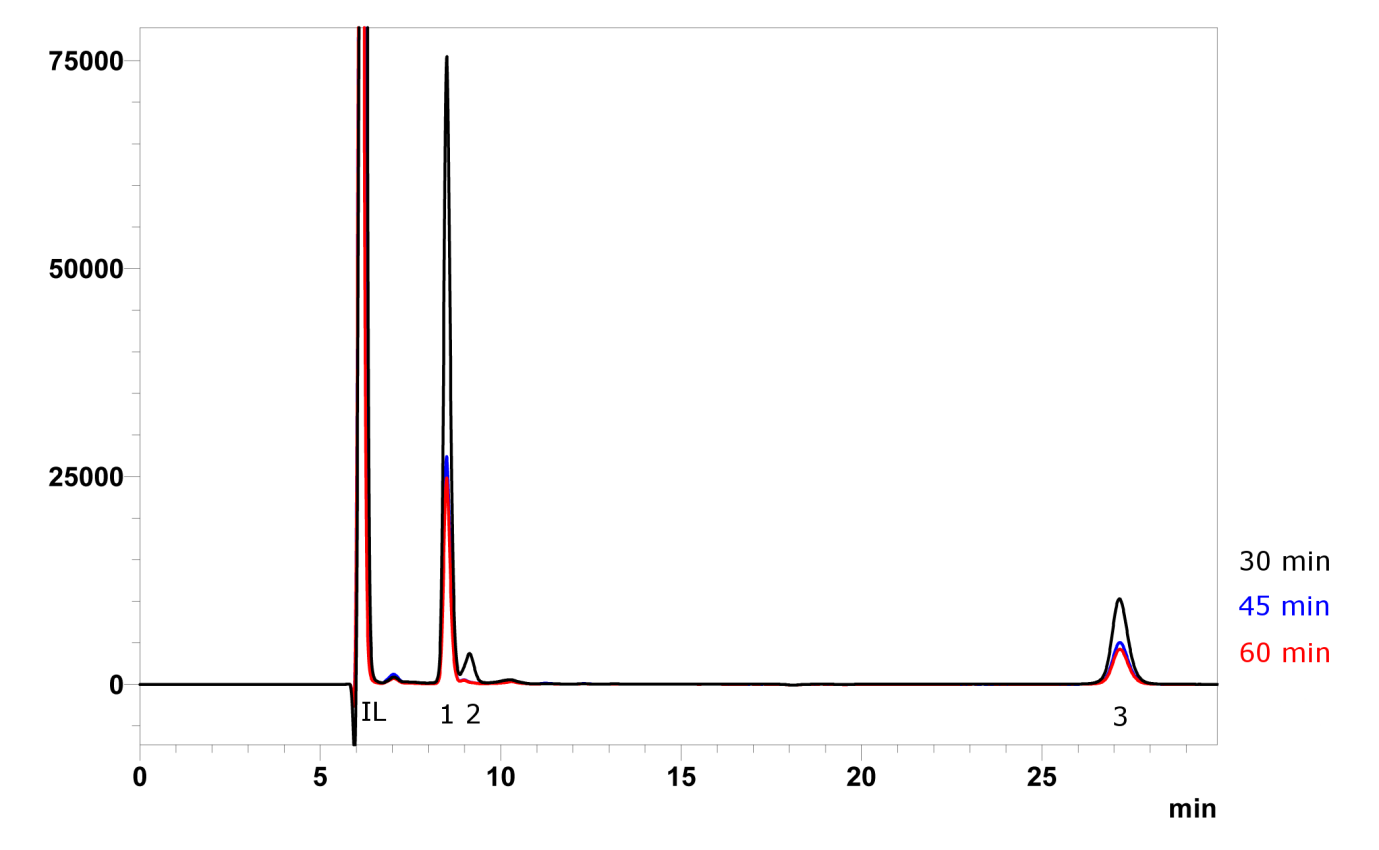

Supplement: S2 Fig — Peak 1 –glucose; peak 2 –fructose; peak 3—HMF. The trace is from the RI detectorwhich shows both HMF and the sugars. The UV/vis trace was used for quantification of HMF due to its higher sensitivity. (TIF) [file pone.0163835.s002.tif]

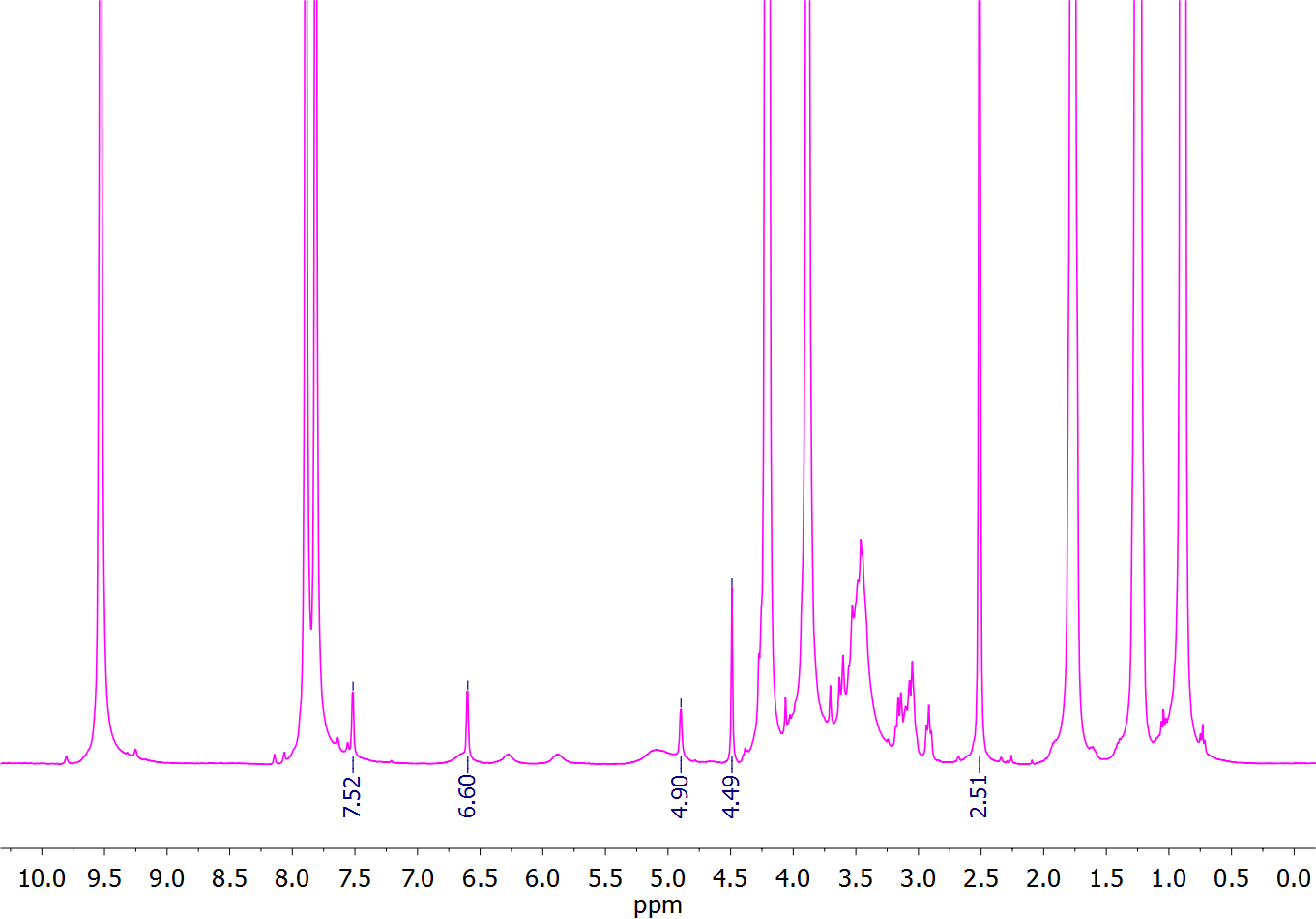

Supplement: S3 Fig — The mixture contained 0.1 g glucose, 0.7 g [C4C1im]Cl, 8.1 mol% CrCl3.6H2O. HMF signals appear at 7.5, 6.6, 4.9 and 4.5 ppm. (TIF) [file pone.0163835.s003.tif]

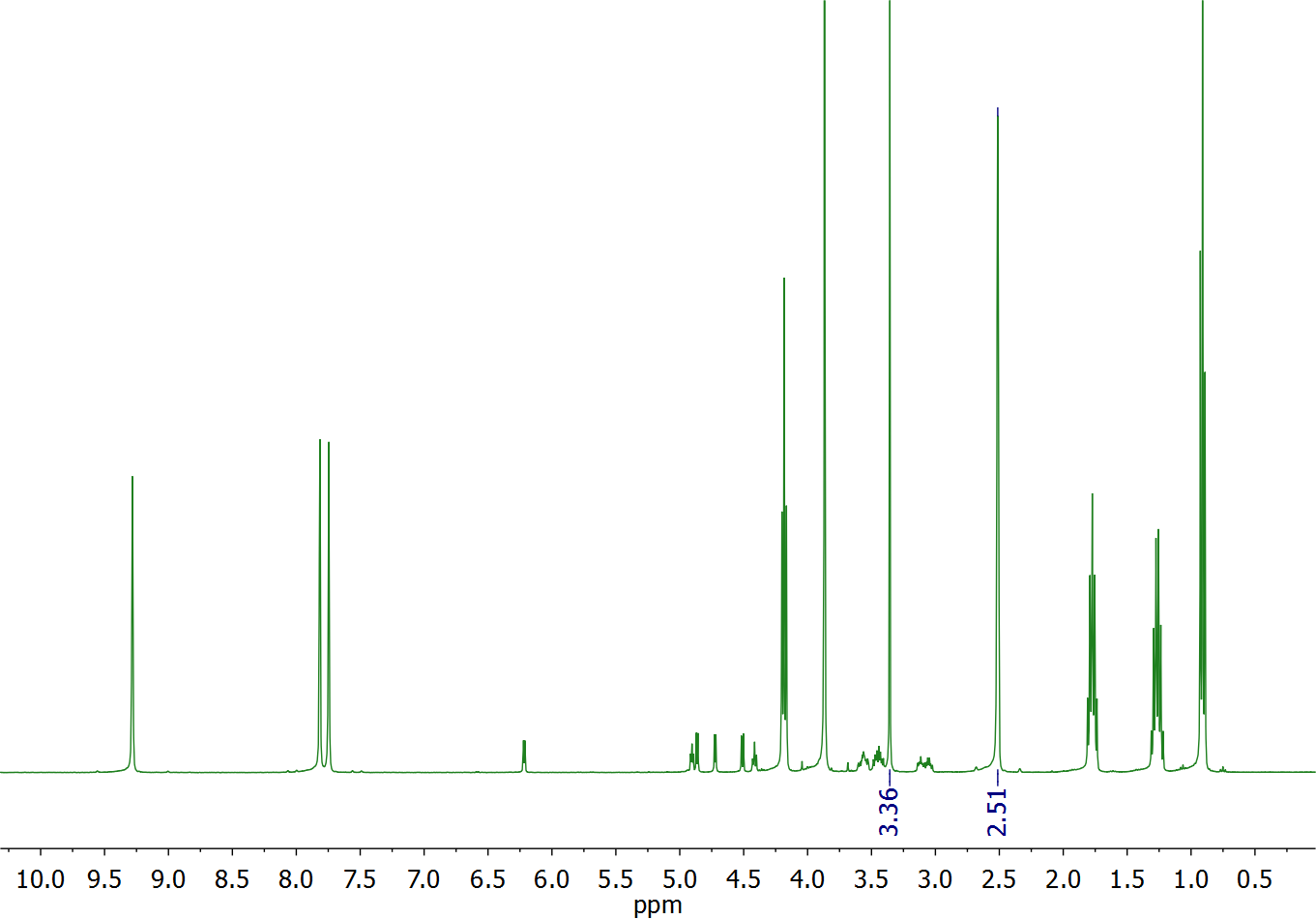

Supplement: S4 Fig — (TIF) [file pone.0163835.s004.tif]

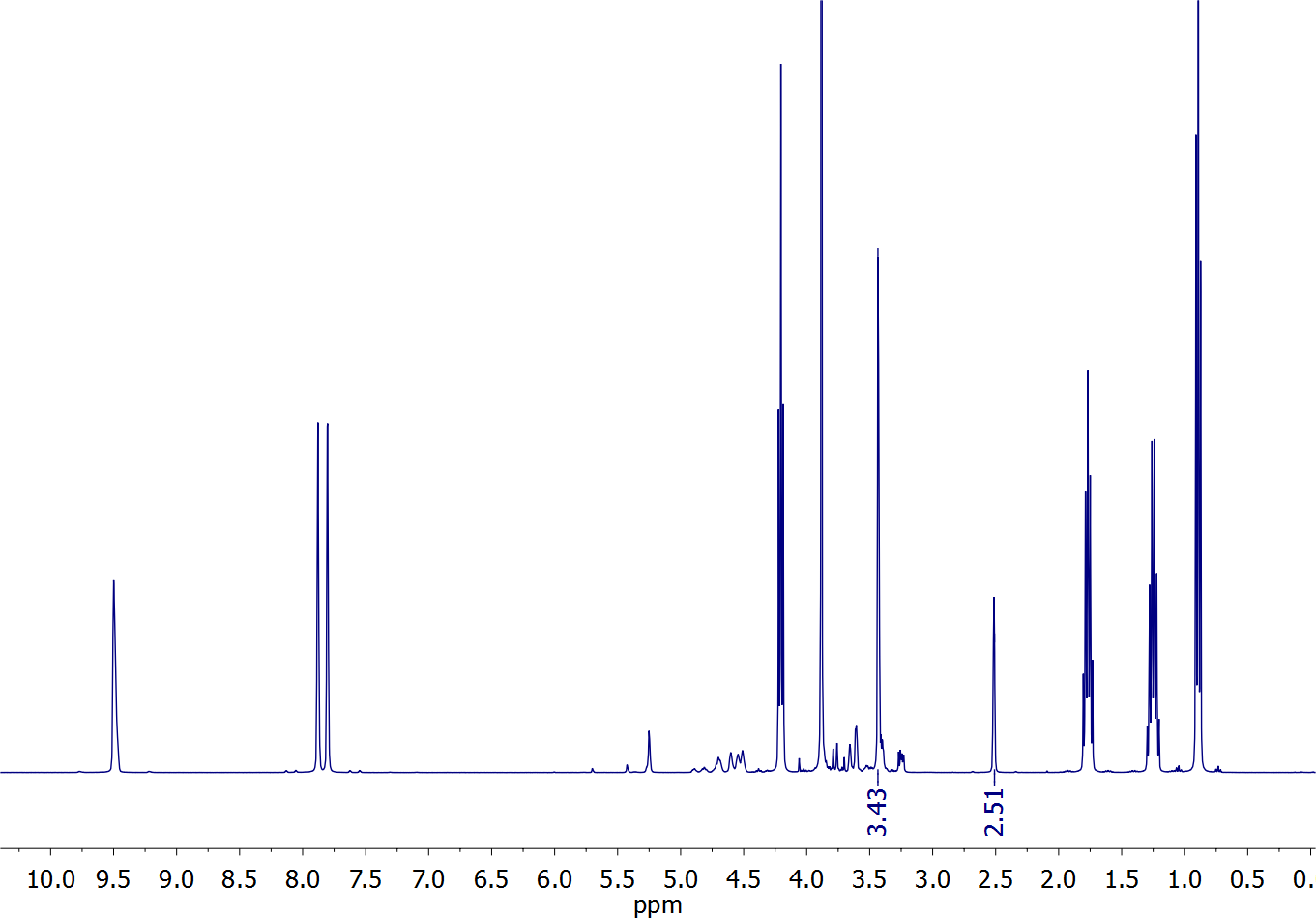

Supplement: S5 Fig — (TIF) [file pone.0163835.s005.tif]

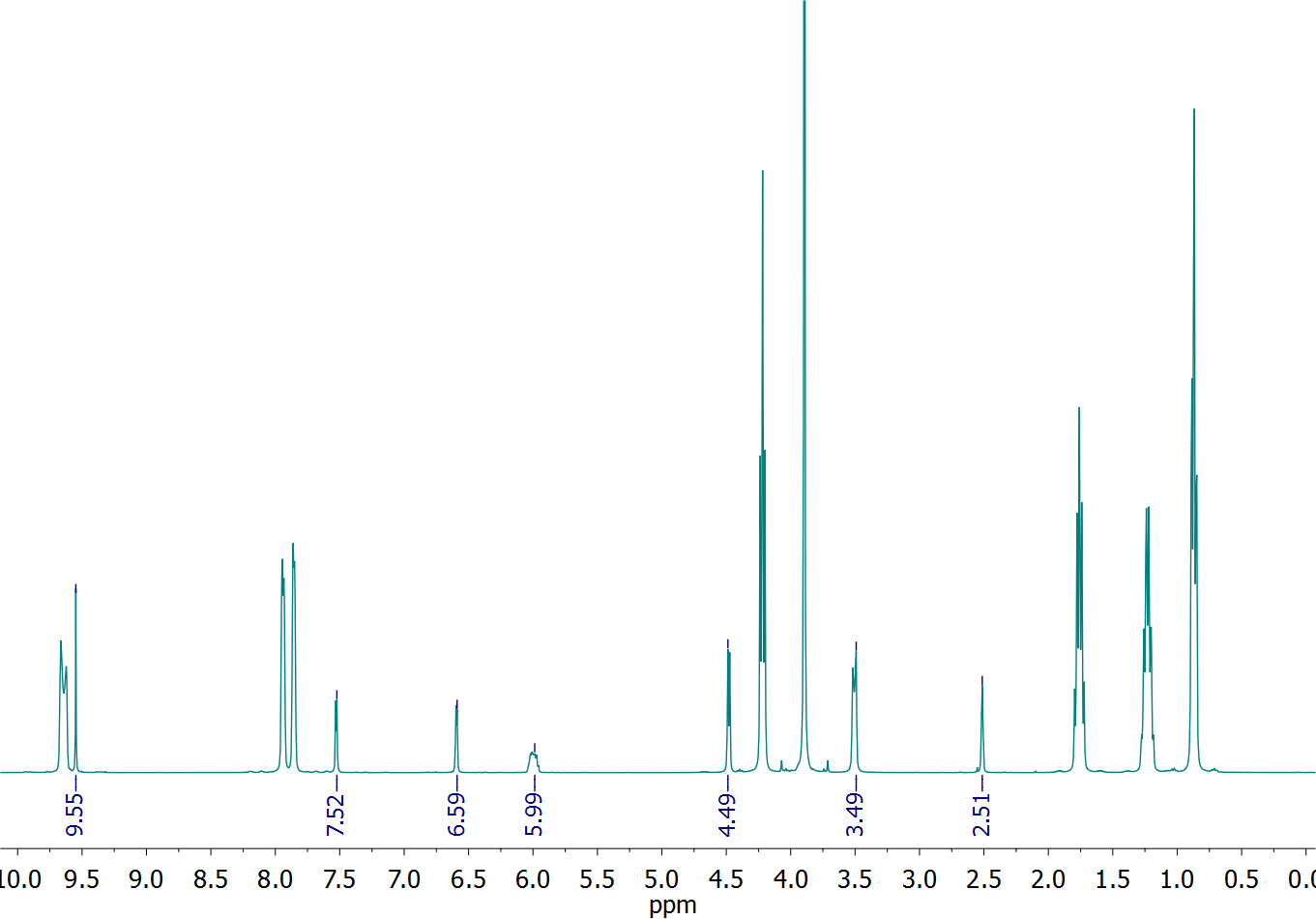

Supplement: S6 Fig — (TIF) [file pone.0163835.s006.tif]

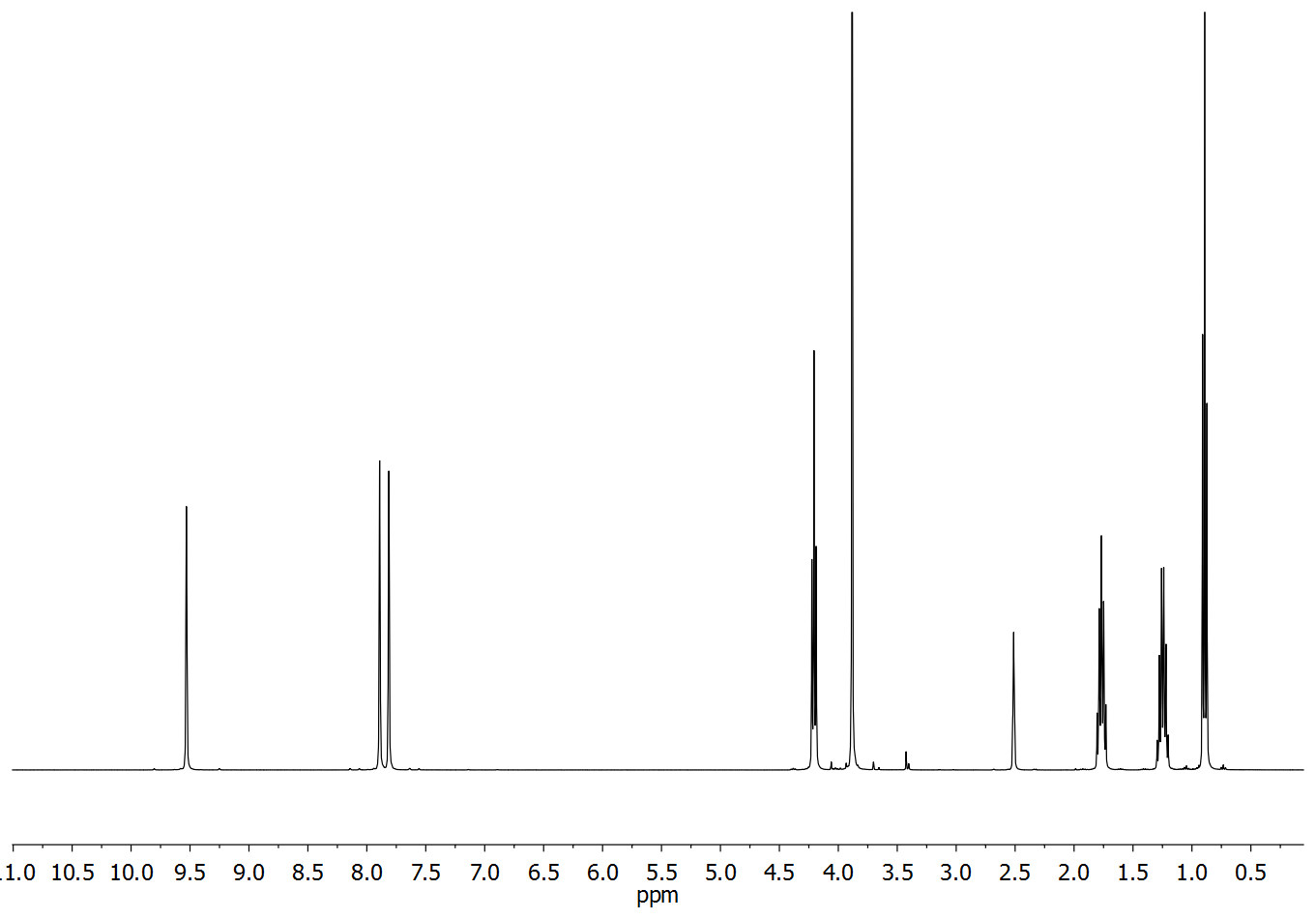

Supplement: S7 Fig — (TIF) [file pone.0163835.s007.tif]
